# Unveiling pseudo-pulseless electrical activity (pseudo-PEA) in ultrasound-integrated infant resuscitation

**DOI:** 10.1007/s00431-023-05199-3

**Published:** 2023-09-19

**Authors:** Belinda Chan, Susan Sieg, Yogen Singh

**Affiliations:** 1 Department of Pediatrics, Division of Neonatology, University of Utah, Salt Lake City, UT 84108 USA; 2 Department of Radiology and Imaging Science, University of Utah, Salt Lake City, UT 84108 USA; 3 Intermountain Healthcare, Salt Lake City, UT 84108 USA; 4 Department of Pediatrics, Division of Neonatology, Loma Linda University School of Medicine, 11175 Coleman Pavilion, Campus Street, Loma Linda, CA 92354 USA; 5 Department of Pediatrics, Division of Neonatology, University of Southern California, Los Angeles, CA 90089 USA

**Keywords:** Resuscitation, Infant, Pulseless electrical activity (PEA), Pseudo-PEA, Point-of-care ultrasound (POCUS)

## Abstract

Point-of-care ultrasound (POCUS) holds immense potential to manage critically deteriorating infants within the neonatal intensive care unit (NICU) and is increasingly used in neonatal clinical practice worldwide. Recent ultrasound-based protocols such as the Sonographic Assessment of liFe-threatening Emergencies-Revised (SAFE-R) and Crashing Neonate Protocol (CNP) offer step-by-step guidance for diagnosing and addressing reversible causes of cardiorespiratory collapse. Traditionally, pulseless electrical activity (PEA) has been diagnosed solely based on absent pulses on clinical examination, disregarding myocardial activity. However, integrating POCUS into resuscitation unveils the concept of pseudo-PEA, where cardiac motion activity is observed visually on the ultrasound but fails to generate a detectable pulse due to inadequate cardiac output. Paradoxically, existing neonatal resuscitation protocols lack directives for identifying and effectively leveraging pseudo-PEA insights in infants, limiting their potential to enhance outcomes. Pseudo-PEA is extensively described in adult literature owing to routine POCUS use in resuscitation. This review article comprehensively evaluates the adult pseudo-PEA literature to glean insights adaptable to neonatal care. Additionally, we propose a simple strategy to integrate POCUS during neonatal resuscitation, especially in infants who do not respond to routine measures.

*Conclusion*: Pseudo-PDA is a newly recognized diagnosis in infants with the use of POCUS during resuscitation. This article highlights the importance of cross-disciplinary learning in tackling emerging challenges within neonatal medicine.

**Table:** 

**What is known:** *• Point-of-Care ultrasound (POCUS) benefits adult cardiac arrest management, particularly in distinguishing true Pulseless Electrical Activity (PEA) from pseudo-PEA.* *• Pseudo-PEA is when myocardial motion can be seen on ultrasound but fails to generate palpable pulses or sustain circulation despite evident cardiac electrical activity.*	
**What is new:** *• Discuss recognition and management of pseudo-PEA in infants.* *• A proposed algorithm to integrate POCUS into active neonatal cardiopulmonary resuscitation (CPR) procedures.*

**Supplementary Information:**

The online version contains supplementary material available at 10.1007/s00431-023-05199-3.

## Introduction

Cardiac arrest affects approximately 13 out of 1000 hospital admissions of infants and children and continues to have a high mortality rate of up to 59% [1]. Among the recognizable cardiac rhythms during these events, pulseless electrical activity (PEA) is commonly observed in the clinical practice. PEA is characterized by visible cardiac electrical activity on an electrocardiogram (ECG) when there is no detectable pulse on clinical examination [2]. The prompt identification and treatment of reversible causes of PEA are critical to achieving the return of spontaneous circulation (ROSC) and improving outcomes. Point-of-care ultrasound (POCUS) has recently emerged as a rapid diagnostic tool for discerning reversible PEA conditions like hypovolemia, tension pneumothorax, cardiogenic shock, and cardiac tamponade. Integrating POCUS into resuscitation procedures is a well-established practice in adult emergency medicine [3]. POCUS has been reported to unveil cardiac motion in 10–35% of adults initially classified as experiencing PEA or asystole based solely on ECG readings [4–7]; introducing the concept of pseudo-PEA, which is defined by observable myocardial motion activity on ultrasound but insufficient to generate a palpable pulse. In adults, pseudo-PEA is managed and prognosticated differently from true PEA, which is without cardiac motion [4]. However, pseudo-PEA has never been described in infants and children prior to using POCUS in resuscitation routinely.

In recent times, the utility of POCUS has extended into neonatal resuscitation. Protocols like the Sonographic Assessment of liFe-threatening Emergencies-Revised (SAFE-R) and Crashing Neonate Protocol (CNP) provide algorithmic guidance for identifying the causes of cardiorespiratory collapse in infants and target-specific interventions [8, 9]. Although these neonatal POCUS protocols help systematically identify reversible causes of cardiorespiratory collapse, they do not address pseudo-PEA in the neonatal and pediatric population. There would be an anticipated rise in the detection of pseudo-PEA cases due to the increased application of POCUS in collapsed infants enabling cardiac motion visualization. However, currently Pediatric Advanced Life Support (PALS), Newborn Life Support (NLS), and Neonatal Resuscitation Protocol (NRP) provide no guidance in managaing pseudo-PEA and corrdinating POCUS amid active chest compression (CPR) [2, 10, 11].

This review article delves into adult literature for insights into distinguishing true PEA from pseudo-PEA, managing pseudo-PEA, and integrating POCUS into resuscitation protocols. Although the pathophysiology and treatment of PEA may differ between adults and infants, the principles derived from adult knowledge and experience provide a foundation for adapting similar treatment approaches to pseudo-PEA in infants and children. We present a cardiac arrest case of a preterm infant who was diagnosed with pseudo-PEA using POCUS. We propose the ultrasound-integrated infant resuscitation algorithm that can be applied into the clinical practice.

## Defining true PEA and pseudo-PEA

PEA, characterized by organized cardiac electric activity on ECG but lacking a palpable pulse, reflects electro-mechanical dissociation [12]. Siller et al. elucidate its origin, attributing PEA to “severe respiratory failure, metabolic derangements, or mechanical obstruction uncoupling cardiac electrical signals and contractile function” [13]. POCUS has been demonstrated to differentiate true PEA from pseudo-PEA, the former signifying cardiac standstill while the latter represents detectable cardiac motion yet insufficient to yield a pulse on clinical examination [14]. Instead of being separate entities, pseudo-PEA and true PEA are likely to be a continuum of declining cardiac wall motion [15]. The decompensation process in pseudo-PEA involves initially organized weakening contractions that evolves into disarray, akin to agonal twitching [6]. True PEA entails cessation of cardiac motion as hypoxia and acidosis worsens, potentially culminating into asystole and demise [15]. PEA’s dynamic and distinct pathophysiology contrasts with shockable tachyarrhythmias, which usually have abrupt onset [15, 16].

## Incidence of true PEA and pseudo-PEA

PEA’s prevalence in adult cardiac arrests ranges from 19 to 23% [6, 16]. Integrating cardiac POCUS into adult resuscitation protocols has shown discernible contractions in 10–35% of cases initially thought to be asystole [6, 7, 10–12]. In a cardiac arrest case series, 27% of patients were in PEA with 18% of those exhibiting pseudo-PEA through ultrasound-detected cardiac motion [6]. Significantly, pseudo-PEA is linked to a more favorable prognosis and an increased chances of achieving ROSC compared to true PEA [4, 16]. A recent meta-analysis has shown that pseudo-PEA patients were 4.4 times more likely to attain ROSC than those with cardiac standstill in true PEA (RR 4.35, 95% CI 2.20–8.63, *p* < 0.00001) [16].

## Management approach of true PEA and pseudo-PEA in adult

The management approaches for true PEA and pseudo-PEA are distinct. Pseudo-PEA warrants aggressive resuscitation due to its potential for recovery [4]. After differentiating pseudo-PEA from true PEA based on the observed cardiac motion, clinicians need to prioritize on identifying some of the reversible causes, encompassing the *5* *Hs* (*hypovolemia*, hypoxia, hydrogen ion [acidosis], hyper/hypokalemia, hypothermia) and *5 Ts* (toxins, *tension pneumothorax*, *tamponade [cardiac]*, thrombosis [coronary, myocardial infarction], thrombosis [pulmonary embolus]). POCUS can help in rapidly identifying *hypovolemia*, *tamponade*, *and pneumothorax*. Following early detection and targeted intervention for the reversible cause if present, early use of cardio-active medications could augment cardiac function in pseudo-PEA patients. Studies have shown that pseudo-PEA patients were more responsive to continuous adrenergic inotropes or vasopressin and established ROSC, unlike true PEA cases [6, 17]. POCUS can also aid in monitoring chest compression efficacy and synchronizing chest compressions with cardiac motion in pseudo-PEA [5]. Synchronized systolic compressions yield better aortic pulse pressure and coronary perfusion, improving outcomes in the animal models with pseudo-PEA [18]. No doubt, in general, both true and pseudo-PEA have poor prognoses, especially prolonged true PEA. Still, early identification of underlying etiology and aggressive targeted management improves outcomes, especially in patients with pseudo-PEA [4].

## Integrating POCUS into adult resuscitation

The role of POCUS in the adult CPR practice is well established. It is currently employed routinely during in-hospital cardiac arrests, trauma scenarios in the emergency room, and field resuscitations [5]. Beyond assessing cardiac function and recognizing reversible factors, the use of POCUS plays an important role in checking pulses, establishing chest compression efficacy, synchronizing compressions with cardiac contractility, and monitoring response to interventions [5]. Drawing from the successful integration of POCUS in adult resuscitation, these lessons are invaluable when developing similar protocols for infants and children. Some of the key points to consider:

- Swift, uninterrupted chest compressions are paramount: strategies to avert prolonged pauses include initiating the first CPR cycle while preparing the ultrasound equipment, capturing subcostal POCUS images for a maximum of 10 seconds during chest compression, storing POCUS clips for 4–5 seconds during CPR pause for pulse check, analyzing images in the subsequent CPR cycle, and effectively communicating findings with the team [3, 19].
- Assigning dedicated and trained personnel for POCUS tasks is crucial [20].
- Leveraging Doppler ultrasonography on the femoral or carotid artery enhances pulse checks with speed and precision in adults, as studies have shown that Doppler is superior over manual palpation of pulses during CPR [20].
- Cardiac POCUS is more effective than ECG in detecting cardiac arrhythmias and heart rate, particularly in cases of chest wall edema and motion artifact during chest compression [20].
- Employing multiple echocardiography views, as opposed to a single view, enhances the assessment of cardiac contraction and the prediction of CPR outcomes. While beginning with a subcostal view, supplementary apical and parasternal views can follow if feasible without interrupting resuscitation measures [16].
- POCUS contributes to optimal compression by guiding hand placement over the ventricles to ensure effective compressions, preventing aorta obstruction [21], and synchronizing with cardiac systole.
- Expanding POCUS applications to other organ systems can aid in finding other reversible PEA causes [3].
- Inexperienced POCUS operators might exhibit varied abilities in distinguishing between cardiac standstill and minimal cardiac activity; hence, the most experienced POCUS operators should scan and interpret images in such scenarios [22].

## Pseudo-PEA in infants

In neonatal resuscitation, embracing novel concepts and adapting practices are not unprecedented. In 2015, the Neonatal Resuscitation Program (NRP) recommended ECG integration within the newborn resuscitation algorithm, shedding light on the incidence of PEA, which was previously obscured [10, 11]. Published case series and literature provide insight into PEA occurrences in infants soon after birth [13, 23]. The prevalence of PEA is better documented among continuously monitored hospitalized pediatric patients. Among the pediatric intensive care unit (PICU) and neonatal intensive care unit (NICU) admissions, the prevalence of cardiac arrest was 1–5% with 40–50% of cases affecting infants under 1 year old, of which 40% were attributed to PEA and asystole [24, 25]. However, these published case series lack recognition of pseudo-PEA; hence, the prevalence, risk factors, and prognosis of pseudo-PEA in infants would remain unknown until POCUS has gained widespread usage.

For all forms of PEA, including true and pseudo-PEA, infants without ROSC and non-survivors shared traits such as pre-arrest inotrope use, non-respiratory triggers, and delayed epinephrine administration [26]. Prior inotrope consumption indicated already compromised cardiac function, which lowers the chances of post-arrest recovery. Diminished survival prospects were seen in infants with infections, necrotizing enterocolitis, or cardiac defects. While epinephrine timing during cardiac arrest had no significant effect on survival rates, delayed epinephrine administration correlated with non-survival [26]. Differences in NICU and PICU populations might explain the variance in epinephrine timing. Respiratory causes are predominantly responsible for cardiac arrests in NICU and delivery rooms, directing NRP and NLS focus on ventilation and airway management [27]. PALS prioritize restoring cardiac output, which is the more prominent cause in the PICU, potentially contributing to earlier epinephrine use in the PICU compared to neonatal cardiac arrests in the NICU [26]. NRP and PALS algorithms relied solely on ECG readings, possibly impeding timely identification of reversible causes and distinguishing pseudo-PEA from true PEA. Integrating POCUS into active CPR protocols could help in making resuscitation strategies more effective, and possibly early use of epinephrine in patients with cardiac motion.

## Integrating POCUS into neonatal resuscitation 

POCUS has gained popularity in the NICU setting. It has now been recommended by crashing neonatal protocols (SAFE-R and CNP protocols), advocating its integration into resuscitation protocols, especially in infants who do not respond to routine resuscitation measures. SAFE-R directs the incorporation of ultrasound to promptly diagnose reversible life-threatening conditions encompassing tamponade, tension pneumothorax, massive pleural effusion, critical aortic occlusion, ascites, and severe brain bleed [8]. An analogous international expert consensus within the CNP reinforces POCUS utility [9]. While these protocols excel in rapidly identifying reversible PEA cause. These protocols also fail to address the situation when reversible causes are eliminated, but ROSC is not achieved. The specific guidance on recognizing and management pseudo-PEA is also lacking. The following case scenario illustrates POCUS’s role in neonatal resuscitation, helping conceptualize the proposed ultrasound-integrated CRP algorithm.

## Case Scenario

**Fig. 1:**
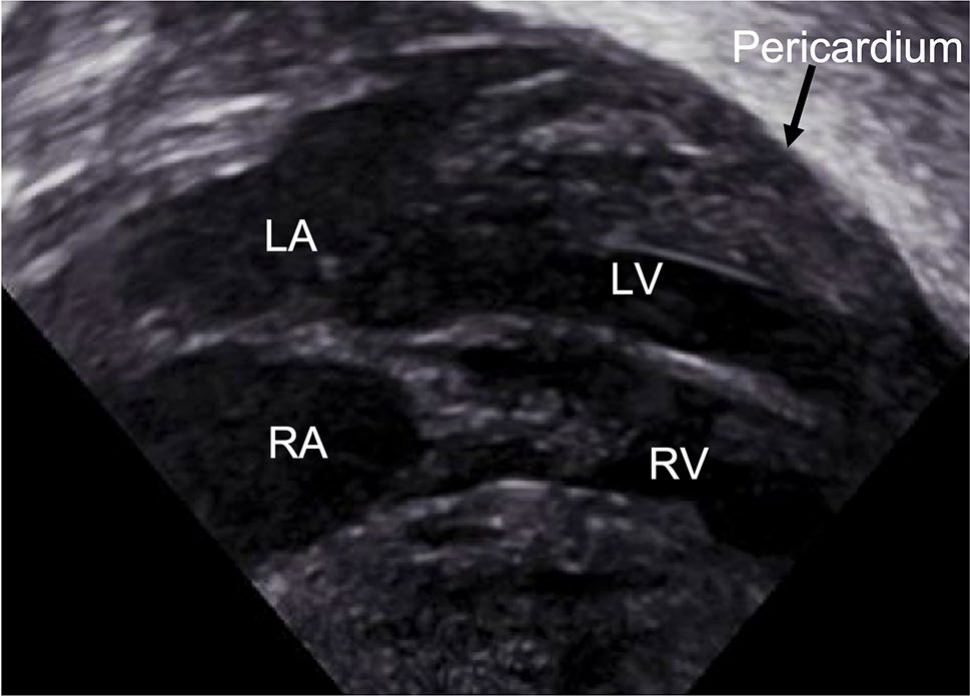
Transverse subcostal view of Cardiac POCUS shows the absence of pericardial effusion or tamponade

A 500 g infant was born at 24 weeks gestational age to a mother who received antibiotics for signs of a urinary tract infection and chorioamnionitis. The infant was delivered following preterm labor, non-reassuring fetal tracing, and difficult birth extraction. After birth, the infant had no respiratory efforts or audible heart rate. After successful intubation, breath sounds were confirmed bilaterally, though no waveform on capnography was detected. Chest compressions were started when the heart rate was noted to be less than 60 bpm with a narrow QRS complex on the ECG. One dose of endotracheal epinephrine was given before an umbilical venous catheter was placed. Once intravenous access was established, the infant received one dose of epinephrine and 10 ml/kg of 0.9% normal saline bolus. The infant remained pulseless, and oxygen saturation was undetectable even after two more rounds of CPR and epinephrine. Once the PEA diagnosis was established, the focus shifted to identifying reversible causes. The rapid POCUS assessment was performed at 20 min of life using the SAFE-R protocol. CPR was paused for 10 s during the imaging. The transverse subcostal view revealed an organized cardiac motion with minimal contractility and no evidence of pericardial effusion or tamponade. ECG monitor showed cardiac electric activity with bradycardia but no palpable pulses despite infrequent spontaneous myocardium motion seen on POCUS (Fig. 1 and Video 1). CPR resumed with additional epinephrine and normal saline bolus was given. Lung and abdominal ultrasounds were performed during CPR, which ruled out pneumothorax and ascites. Cardiac POCUS was repeated 24 min after birth and showed minimal cardiac motion, inadequate for maintaining systemic perfusion to sustain life. Resuscitation was stopped after discussing with the parents and team involved. This case and images showed pseudo-PEA diagnosed in an extremely preterm infant encompassed prolonged CPR and multiple epinephrine doses. Despite these interventions, minimal cardiac contraction was observed through ultrasound which led to the cessation of resuscitation. This highlights the disparity between cardiac mechanical motion and electrical activity in achieving ROSC and POCUS could help with decision-making [2].

## Proposed ultrasound-integrated neonatal CPR algorithm

**Fig. 2:**
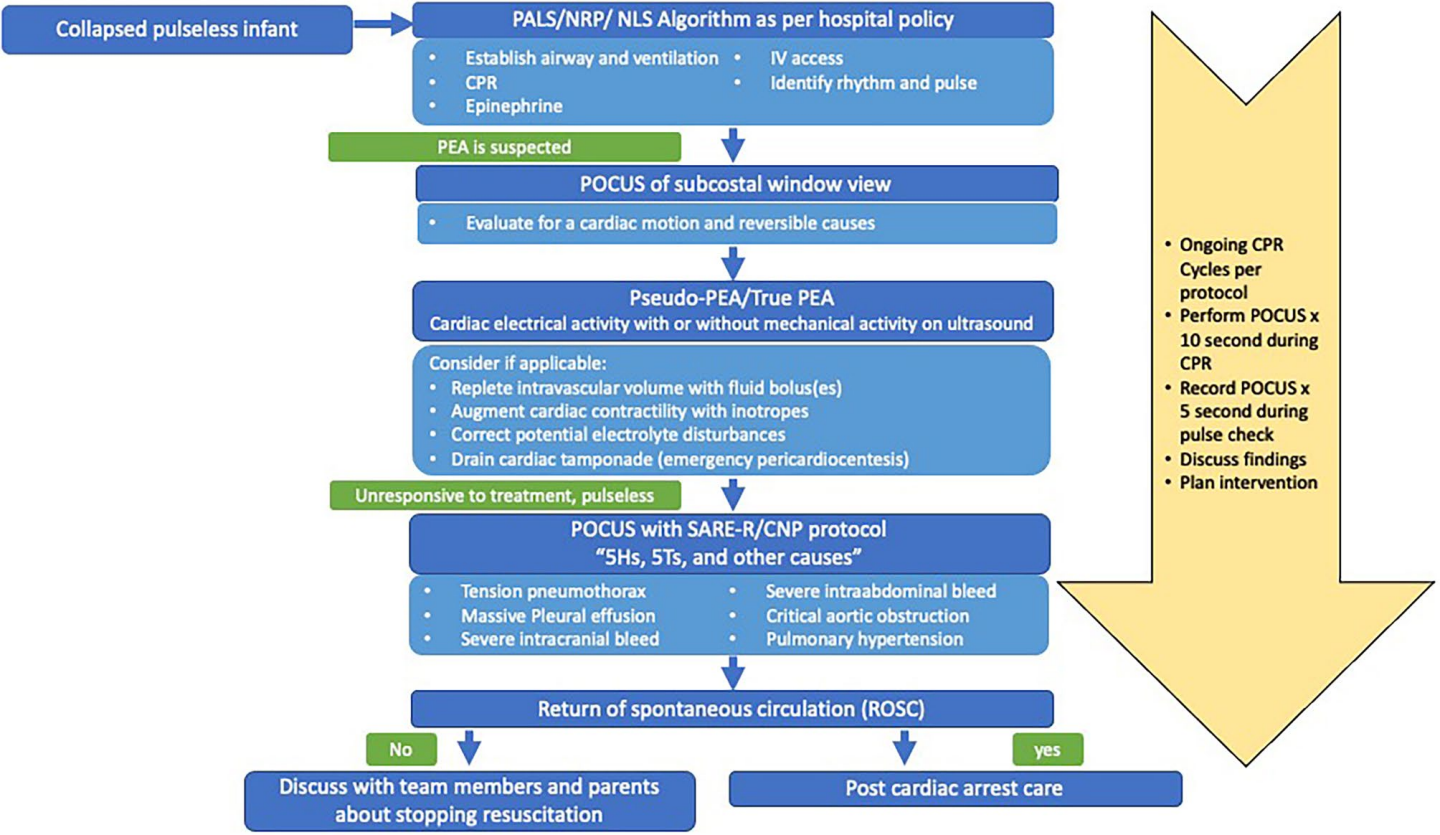
CPR algorithm with POCUS integration

Implementing POCUS during neonatal resuscitation necessitates a clear integration guideline within CPR algorithms and will need more comprehensive discussion among stakeholders. Drawing from the adult POCUS-integrated resuscitation model [3], we propose a systematic approach for POCUS during active CPR (Fig. 2). Initial resuscitation follows NRP/NLS or PALS algorithms, while POCUS assists in differentiating pseudo-PEA from true PEA and identifying reversible causes for infants unresponsive to standard measures. Things to be considered when integrating POCUS into CPR algorithm include POCUS must not obstruct effective CPR or airway management. A skilled POCUS provider rapidly assesses cardiac function for tamponade and myocardial contraction, capturing subcostal window images for 10 s during CPR and storing images for 5 s during pulse checks. Real-time interpretation and communication with the team occur as CPR resumes [19]. If pseudo-PEA is identified, the focus shifts to augmenting cardiac contractility, optimizing ventricular filling, correcting acidosis, or electrolyte imbalances, and addressing cardiac obstructions to enhance perfusion and pulses. In emergency situations such as active CPR, the most effective approach to assessing cardiac contractility on ultrasound is through visual inspection, commonly referred to as “eyeballing”. While objective assessments and measurements of contractility can be performed quickly, they are often less reliable when obtained in suboptimal condition and active CPR. Therefore, qualitative assessment via “eyeballing” is the preferred method, as it was also employed in the given case scenario. Sequential POCUS assessments can help evaluate myocardial response to intervention and assess other organ systems for reversible PEA causes using SAFE-R or CNP protocols. Doppler ultrasonography aids pulse check when obtaining heart rate by manual palpation or pulse oximetry waveform is challenging.

## Decision-making on stopping resuscitation

Despite the best resuscitation efforts, mortality risks and poor neurological outcomes increase with prolonged resuscitation, particularly after 20–30 min of CPR [26, 28]. Best et al. reported no survivors among infants with true PEA [26]. Identifying prolonged true PEA or asystole might justify stopping resuscitation after 20 min of high-quality resuscitation as per NRP and NLS protocols [10, 11]. Determining when to stop CPR in the presence of pseudo-PEA or ineffective cardiac contractions poses significant challenges even to the most experienced clinicians. Decision-making should involve multiple healthcare providers, including attending physicians, nurses, resuscitation team members, and parents [1]. Redirecting to comfort care requires assessing clinical status, cardiac arrest duration, underlying cause, and potential irreversible damage to vital organs, especially the brain. In extremely preterm infants with untreatable PEA cause, extended cardiac arrest, CPR unresponsiveness, and ineligibility for extracorporeal cardiopulmonary resuscitation collectively suggest futility. NRP and NLS guidelines endorse stopping CPR if resuscitation extends beyond 20 min of high-quality resuscitation without ROSC [2, 10, 11, 27]. No guidelines help clinicians determine CPR cessation after prolonged resuscitation without ROSC, even if visualizing insufficient cardiac motion, as in pseudo-PEA. Further studies are needed to guide pseudo-PEA management, especially in infants and young children.

## Conclusion

The growing utilization of POCUS in the NICUs, alongside POCUS-based SAFE-R and CNP protocols, allows the opportunities to adopt this tool during resuscitation, particularly in infants who do not respond to standard resuscitation protocols. As POCUS becomes integral to managing collapsed infants, encounters with pseudo-PEA will become more prevalent. Hence, timely recognition and targeted specific management of PEA is crucial to improve the outcomes. Adult literature indicates different management and improved outcomes with pseudo-PEA as compared to true PEA without cardiac activity on ultrasonography. Further research is needed to provide clear guidance for managing pseudo-PEA conditions in neonatal resuscitation and effectively integrating POCUS into resuscitation protocols.

### Supplementary Information

Below is the link to the electronic supplementary material.

Supplementary file1 (AVI 5801 KB)

## Abbreviations

CPR: Cardiopulmonary resuscitation
CNP: Crashing Neonate Protocol
ECG: Electrocardiogram
NICU: Neonatal intensive care unit
NLS: Newborn Life Support
NRP: Neonatal Resuscitation Protocol
PALS: Pediatric Advanced Life Support
POCUS: Point-of-care ultrasound
ROSC: Return of spontaneous circulation
PEA: Pulseless electrical activity
SAFE-R: Sonographic Assessment of liFe-threatening Emergencies-Revised
